# Mindsight: Diagnostics in Disorders of Consciousness

**DOI:** 10.1155/2012/624724

**Published:** 2012-11-14

**Authors:** P. Guldenmund, J. Stender, L. Heine, S. Laureys

**Affiliations:** ^1^Coma Science group, Cyclotron Research Center and Department of Neurology, University of Liège and University Hospital of Liège, Sart-Tilman B30, 4000 Liège, Belgium; ^2^BRAINlab, Department of Neuroscience and Pharmacology, Panum Institute, University of Copenhagen, 2200 Copenhagen, Denmark

## Abstract

Diagnosis of patients with disorders of consciousness (comprising coma, vegetative state/unresponsive wakefulness syndrome, and minimally conscious state) has long been dependent on unstandardized behavioral tests. The arrival of standardized behavioral tools, and especially the Coma Recovery Scale revised, uncovered a high rate of misdiagnosis. Ancillary techniques, such as brain imaging and electrophysiological examinations, are ever more often being deployed to aid in the search for remaining consciousness. They are used to look for brain activity patterns similar to those found in healthy controls. The development of portable and cheaper devices will make these techniques more widely available.

## 1. Introduction

**Figure 1:**
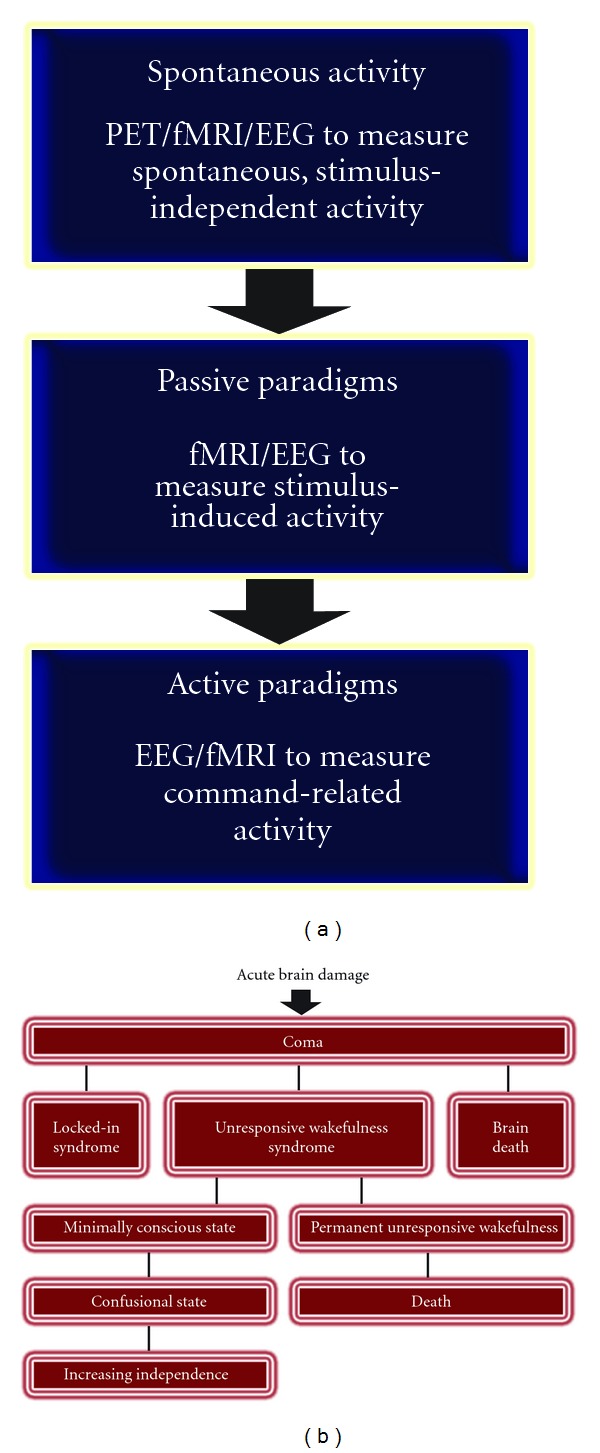
(a) Chronological order of diagnostic methodology. (b) Flow chart of disorders of consciousness.

Recent advances in medicine have led to an increase in survival rate after severe brain damage [1]. Many of the survivors pass into states of disorders of consciousness (DOC), such as coma, unresponsive wakefulness syndrome (UWS, previously known as vegetative state (VS); [2]), or the minimally conscious state (MCS) (Figure 1). In this paper, we will discuss the differential diagnosis of these patients using clinical and ancillary methods. Coma is defined as absence of both arousal and responsiveness and usually lasts no longer than four weeks. Patients in a coma can recover or transit to the VS/UWS or MCS or can become locked-in: a state of near-complete body paralysis in which consciousness is fully recovered but possibilities for communication are generally restricted to eye movement and blinking [3]. In contrast, VS/UWS patients show no behavioral sign of awareness, but have retained some level of arousal. They show eye-opening, have sleep-wake cycles, and are able to maintain vital functions unassisted [4]. VS/UWS patients may show reflex responses to tactile, pain, auditive, or visual stimuli, but fail to show response to commands. The recent renaming of the “vegetative state” into UWS has multiple reasons. Firstly, UWS gives a better description of the behavior profile in which the patient is awake, but nonresponsive. Secondly, it has less negative connotation, as a vegetative state could be associated with the patient being “vegetable-like” [2]. The VS/UWS can be a permanent state or can evolve to the MCS. The MCS is characterized by inconsistent, nonreflexive behavior indicating a form of fluctuating low-level awareness [5]. Brain imaging techniques and behavioral assessments indicate that these patients do feel pain and emotions, as will be discussed later in this review. The MCS has recently been stratified into MCS+ (plus) and MCS− (minus), based on the complexity of behavioral responses. MCS− refers to patients only showing minimal levels of behavioral interaction and nonreflexive movements. They may show orientation to noxious stimuli or visual pursuit of moving or salient stimuli. Environmental stimuli may elicit appropriate affectional responses, such as crying or smiling triggered by familiar voices or faces [6]. MCS+ is characterized by more complex behaviors such as command following, language apprehension, intelligible verbalization, or verbal or gestural yes/no responses [7].

Making the distinction between MCS and VS/UWS patients is a perplexing clinical problem. The clinical presentations of the patients can be relatively similar, although they may have significantly different levels of awareness. The differential diagnosis between the two states is important for prognostic, therapeutic, and ethical reasons. The prognosis of MCS is thought to be relatively better than that of VS/UWS [8, 9]. Giacino and Kalamar [10] showed that 12 months after brain injury, about half of patients in the MCS showed “no or moderate” disability scores as assessed by the Disability Rating Scale, compared with only 3% of the patients in the VS/UWS. Following posttraumatic MCS patients for five years after injury, Lammi et al. [11] reported similar recovery rates within the first year. They also noted that one-third of the patients recovered to a level of independent functioning within two and five years after injury. 

Besides prognosis, a key question is the potential for suffering in the two states. Patients in the MCS may show oriented behavioral pain responses, while those in the VS/UWS do not. Whether the pain reaction in the MCS is purely reflexive or has a subjective component is not clear, but evidence from neuroimaging studies suggests the latter [12]. It is reasonable to assume that interventions such as social and physical care and adapted analgesia treatment may help to improve the quality of life and alleviate suffering of these patients. However, such care is resource consuming, emotionally taxing on the relatives, and may be considered futile. It might therefore be neglected in cases of VS/UWS where the prospects of awakening are poor.

Decisions of life and death are difficult but unavoidable in DOC. The ethical framework of these decisions is incompletely developed, and most life-or-death decisions are taken on a case-by-case basis and show major culture-dependent differences [13]. Recent studies have indicated that our conception of possible suffering in these patients may play a decisive role in this regard. A European survey showed that most of the healthcare providers who think a patient in the VS/UWS does not feel pain, agree with treatment withdrawal for these patients [14]. Legal precedence from a series of much debated cases (mainly in the US, and UK) has established the possibility to withdraw artificial life support in the chronic phase of VS/UWS [15]. In contrast, a recent verdict by a British court established the right of a persistent minimally conscious patient to retain life [16]. In short, the differential diagnosis between the MCS and VS/UWS may have dramatic therapeutic, legal, and social consequences for the patient. This diagnosis is usually passed using bedside clinical methods.

## 2. Clinical Assessment

Consciousness is clinically described as consisting of two major dimensions: arousal and awareness. Arousal is the level of alertness, while awareness is the “content” of consciousness. Awareness can in turn be divided into awareness of the external world and awareness of the internal world (i.e., stimulus-independent thoughts, mental imagery, inner speech, etc. [18]). Arousal can be assessed behaviorally by examining the presence of spontaneous or stimulus-induced eye-opening. Quantification of awareness through behavioral assessment relies on discriminating between automatic responses and nonreflex oriented movements or response to command. Motor responses can be inconsistent, very small, and easily exhausted [19], making such examinations challenging. It is important to use examinations that do not exhaust the patient to a level where he or she loses the ability to cooperate. For example, visual pursuit is one of the behaviors distinguishing MCS from VS. Visual stimuli with emotional importance to the patients—as for example using a mirror—provide better results. An explanation for this is that the autoreferential value of our own image captures our attention and gives rise to a sense of self-awareness [20]. It has to be kept in mind that although visual pursuit is associated with consciousness, a lack of visual pursuit does not indicate unconsciousness. 

Several studies have shown an alarmingly high diagnosis error rate of 30–40% using nonstandardized consensus-based techniques in DOC [21]. Hence, for reliable bedside diagnosing, use of systematized scoring systems is mandatory [6, 21]. Most standardized behavioral scales are relatively easy to use and do not rely on expensive or specialized equipment, making it possible for caretakers to apply them in both hospitals and nursing homes. Recently, the American Congress of Rehabilitation Medicine conducted an evidence-based review of behavioral assessment scales for DOC, providing recommendations for clinical use founded on content validity (i.e., to what degree scale items cover a representative sample of DOC-relevant behavior), reliability, diagnostic validity, and ability to predict functional outcomes [22]. The authors concluded that the Coma Recovery Scale revised (CRS-r) [6] is the most reliable tool for differential diagnosis of DOC. 

The CRS-r was developed to differentiate VS/UWS from MCS and to identify patients that have emerged from MCS in the subacute and chronic state. It consists of six subscales measuring auditory, visual, motor, and verbal functions, communication and arousal. The first five subscales go from willful, consistent functional responses to no measurable response or reflexive activity. The arousal subscale indicates the level of arousal ranging from attention through eye-opening to none. The total CRS-r scores range from 0 (worst) to 23 (best) and are based on specific behavioral responses to sensory stimuli [6] (see Table 1 for a detailed description of the scale). The CRS-r has excellent content validity and is the only scale which includes all of the Aspen Workgroup criteria for good standardized administration and scoring [5]. It showed good interrater reliability, test-retest reliability, and internal consistency. 

An alternative to the CRS-r is the Full Outline of Unresponsiveness score (FOUR). According to the American Congress of Rehabilitation Medicine study, this scale has good interrater reliability [23]. It uses four subscales: eye response, motor response, respiration, and brainstem reflexes, with each subscale having a maximum score of four. The FOUR was developed for the acute setting, has excellent internal consistency, and showed to be predictive of good recovery versus disability or death at one-month after injury if administered within the first 24 hours after brain injury. Unfortunately, this scale has ill-defined standardization of administration and scoring and does not cover all of the Aspen Workgroup criteria for MCS (low content validity). Use of the FOUR scale for differential diagnosis of VS/UWS-MCS is therefore not recommended [22, 24, 25].

The problem of pain assessment is of equally great importance, both in the (sub)acute and chronic phases. Patients in MCS show cerebral correlates of pain processing similar to healthy awake people and these activations are more widespread than in VS/UWS [12]. This indicates the potential for suffering of patients in MCS. Patients with a DOC usually cannot willfully communicate experiencing pain, but the patients may show clinical signs of suffering. Although a spontaneous facial expression (e.g., a grimace or a cry) by itself does not imply much, its occurrence after a noxious stimulation does suggest that the patient can feel pain. While it cannot be proven that behavior or brain activations actually correspond to a subjective experience of pain, the possibility that the patient may be suffering should in itself be reason enough for considerations of palliative treatment. The Nociception Coma Scale was developed to assess the behavioral signs of pain in patients to address the possible need for analgesic treatment. The scale has demonstrated a good interrater agreement, concurrent validity and sensitivity [21, 26, 27]. A new version is under development: the Nociception Coma Scale revised [28]. It evaluates motor, verbal, and facial responses after noxious stimulation and can better discriminate between pain and no pain. The scale ranges from zero to nine (see Table 2), in which a score of four or higher indicates that the patient is likely to feel pain, making analgesic treatment necessary. The Nociception Coma Scale is currently the most informative tool available. However, the potential for false negative responses (e.g., in paralyzed patients) and our limited knowledge of pain perception in this group of patients indicate a venue of further research, combining behavioral and neuroimaging tools. 

Clinical assessment is a first and important step towards a proper diagnosis. Unfortunately, behavioral assessment contains several pitfalls, making it problematic as a stand-alone tool. Discriminating between reflexive and willful behavior can be notoriously difficult: for instance, determining whether an eye-blink or small limb movement represents an “automatic” reflex or a “willed” consciously controlled movement may be subjective and challenging. To avoid misinterpretation, three out of four repetitions of each task are generally required for a positive result using the CRS-r, but the risk of false positives cannot be ruled out. Moreover, several sources of false negative results exist. These include problems with brain arousal and attention (i.e., the fluctuating level of consciousness in MCS patients, brainstem lesions, seizures, sedative or anti-epilepsy or antispasticity drugs), sensory and motor output (i.e., aphasia, apraxia or deafness [29, 30]), language comprehension, restraining and immobilizing techniques, and pain [26]. Therefore, absence of adequate response to command using traditional bedside methods does not necessarily prove a patient is unconscious. Finally, due to lack of a diagnostic gold standard, criterion validity and diagnostic value (i.e., the scale's ability to establish an accurate diagnosis compared with the true diagnosis as measured by a reference standard) cannot be determined for any available scoring system [22]. 

Recent improvements in imaging-based diagnostic methods may complement the clinical scoring systems, minimizing the risk of taking therapeutic decisions on erroneous diagnostic backgrounds. As we will see, neuroimaging methods can complement clinical assessment by looking at task-dependent and task-independent brain activations. These can be compared to those observed in conscious healthy controls. 

## 3. Ancillary Testing

### 3.1. Positron Emission Tomography

Fluorodeoxyglucose positron emission tomography (FDG-PET) provides a proxy measure of cerebral glucose consumption, thereby giving an approximation to functional tissue integrity. This can accompany structural estimates of brain damage provided by other brain scan methods [31]. In acute coma, global brain metabolism is reduced to about 50–70% of normal [32]. The average global cerebral metabolic rate of glucose in VS/UWS is about 40–50% of normal values, but may decrease to 30–40% over time [33].

**Figure 2:**
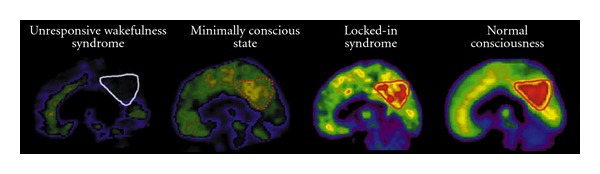
Spontaneous brain activity in VS/UWS, MCS, locked-in syndrome, and health, as seen with PET. A triangle is drawn around the precuneus; an area whose spontaneous metabolic intensity is indicative of the level of consciousness (adapted from [17]).

Several studies have demonstrated that patients in different states of consciousness have distinct *regional* patterns of cerebral metabolic dysfunction. These pattern differences can potentially serve as landmarks for differential diagnosis. Patients in VS/UWS show relative metabolic preservation of the brainstem, probably relating to their preserved functions of arousal and wakefulness [17]. Compared with VS/UWS, patients in MCS show higher activity in the precuneus and less widespread cortical hypometabolism [17]. Patients in MCS and VS/UWS show relative decreases of metabolism in the frontoparietal networks, comprising the medial prefrontal, precuneal, and lateral temporoparietal cortex, as well as the thalamus, compared to healthy controls (Figure 2) [17, 34]. These areas overlap with those of the so-called default mode network, which is a pattern of brain regions that show similar dynamics of their spontaneous neuronal activity and has been associated with internal awareness and stimulus-independent thought [35]. In a study by Bruno et al. [7], CRS-r total scores showed a linear correlation with metabolism in default mode network regions. Furthermore, regions of the external control network (a.k.a. extrinsic control network), encompassing left and right lateral parietal and lateral prefrontal cortices, have been found to be more hypometabolic in VS/UWS than in MCS and normal consciousness [36]. These areas are important for awareness of the external world [18].

Phillips et al. [38] demonstrated the possibility of using a computerized user-independent learning algorithm to stratify patient groups by level of consciousness, thereby calculating the probability that a DOC patient is “unconscious” VS/UWS or “conscious” MCS or locked-in. Recently, studies with FDG-PET have also found differences in brain metabolism between MCS+ and MCS−, showing a relative hypometabolism in left hemispheric language areas in MCS− patients, indicating that this group could suffer from different degrees of aphasia [7]. 

Early DOC brain activation studies, measuring blood flow using H_2_O-PET, showed that auditory stimulation leads to activation in primary and associative auditory cortices more in healthy controls and MCS patients than in VS/UWS patients [39]. Interestingly, brain activation in MCS was increased more as a response to emotionally meaningful sounds (a baby crying, storytelling by a familiar voice) than to meaningless sounds [39–41]. A main distinction between MCS and VS/UWS, related to the level of consciousness, is the potential for pain perception. A limited but growing field of pain research showed that MCS patients do feel pain [26]. Pain induction in healthy controls and MCS patients is followed by activation in a widespread cortical and subcortical pain matrix [12], while VS/UWS patients only show activation restricted to lower-level subcortical and primary cortical areas [42]. 

### 3.2. Magnetic Resonance Imaging

Magnetic resonance imaging (MRI) is used to acquire detailed insight into the extent of structural brain damage suffered by a DOC patient and is now frequently replacing traditional computer tomography techniques. MRI has higher spatial resolution, does not expose the patient to radiation, and is becoming increasingly available. However, an MRI scan is still not always easy to obtain in noncollaborative patients with DOC. Although there is yet no general understanding of how specific brain damage correlates with the level of consciousness in a DOC patient, certain types of structural brain damage can be linked to prognosis. Pontine, basal gangliar and midbrain damage have been associated with poor chances of recovery, and can be detected using MRI techniques [43]. 

Functional MRI is a technique developed to visualize brain activation patterns and has multiple applications, such as “resting state” fMRI, which is a powerful addition to the neuroimaging arsenal as a task-free imaging protocol. This method looks at oscillating patterns of spontaneous neuronal activity at the low-frequency range, visualizing networks of brain areas that show similar spontaneous activity (i.e., functional connectivity). Of particular interest for the assessment of DOC patients is the default mode network, the integrity of which has been shown to correlate to the level of consciousness in a subject (for a review, see [44]). Vanhaudenhuyse et al. [45] have shown that at the group level, connectivity of the precuneus within the default mode network disintegrates when proceeding from normal consciousness to MCS, VS/UWS, and coma. However, its diagnostic power at the single subject level remains to be shown. Moreover, Norton et al. [46] observed an intact default mode network in two coma patients that later recovered consciousness. This study shows that the presence of a default mode network does not prove a patient is conscious, although it may serve as a predictor of good outcome.

fMRI activation studies have confirmed the previously discussed PET activation studies, showing that auditory, visual, and somatosensory activation is more restricted to lower sensory regions in VS/UWS as compared to the more widespread cortical activation seen in MCS [47–51]. For example, Qin et al. [52] showed that the anterior cingulate cortex (part of the limbic emotional network) shows activation during presentation of a patients' own name, in a way that correlates with patients' level of consciousness.

fMRI can also be used to show voluntarily modulated, motor-independent responses to simple commands [53]. Rodriguez Moreno et al. [54] asked patients diagnosed as being in the MCS and VS/UWS to name pictures while lying in an MRI scanner. All MCS patients, and two VS/UWS patients, showed complete or partial activation of the object-naming brain network. Bekinschtein et al. [55] have shown that two patients previously diagnosed as being at the border between VS/UWS and MCS responded to the task to move their left or right hand by activating the dorsal premotor cortex contralateral to the instructed hand. This indicates that those patients were trying to move their hand, although this was not translated into motor activity. 

**Figure 3:**
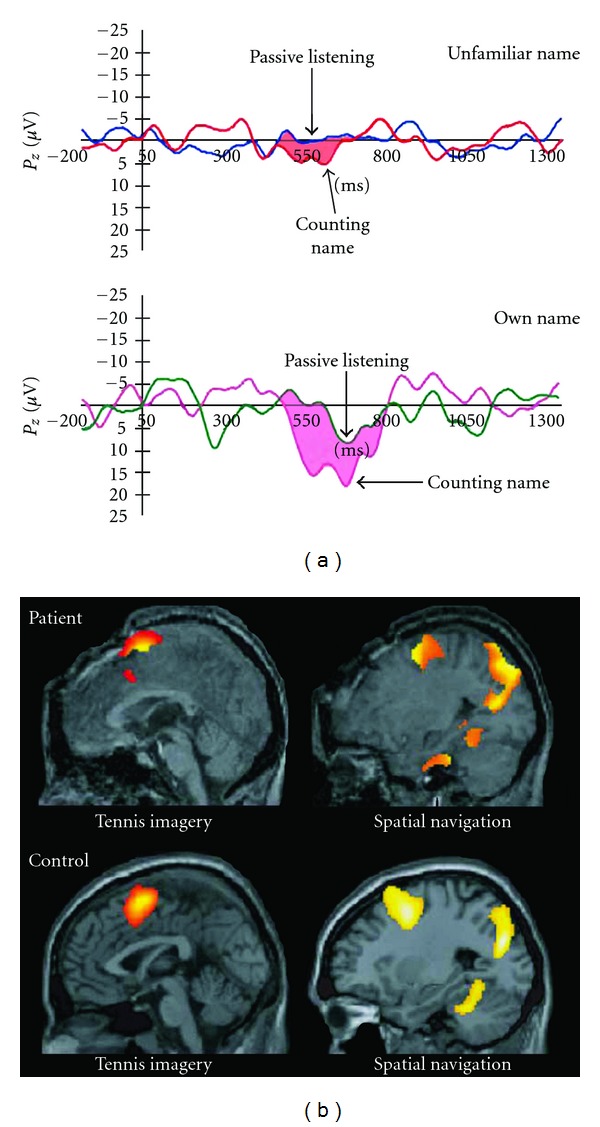
Active and passive paradigms. (a) Differences in event-related response between passive listening to an auditory train of names and actively counting the occurrence of a specific name. Signals are strongest when the patient is counting (active paradigm) its own name. (b) Answering “yes” or “no” by mental imagery using fMRI. Thinking of playing tennis (to answer “yes”) activated motor areas, while thinking of walking through the house (to answer “no”) activated parahippocampal areas (adapted from [19, 37]).

Owen et al. [56] pioneered an active fMRI paradigm in which healthy control subjects and a DOC patient were asked to perform two mental imagery tasks: “imagine playing tennis” and “imagine walking through your own house.” In healthy control subjects, performing the first task activated motor areas, while the second task activated parahippocampal areas (Figure 3). The patient previously diagnosed as VS/UWS consistently showed brain activation patterns similar to those seen in healthy control subjects, implying that the diagnosis based on behavioral tests had been false. Monti et al. [19] have used this mental imagery technique to detect willful modulation of brain activity in five DOC patients, of which only three showed signs of awareness in extensive behavioral tests after the fMRI scan. Furthermore, the authors managed to establish a form of communication with one patient that was initially diagnosed as VS/UWS (but later shown to be behaviorally MCS). This patient was able to answer five out of six questions correctly using “yes” (play tennis) or “no” (walk through your house) answers. However, in a similar mental imagery experiment by Bardin et al. [57], two MCS patients incapable of communicating using the mental imagery paradigm, were able to communicate outside the scanner (one subject via head movements, the other verbally). 

Diffusion tensor imaging is a relatively recent addition to the analysis of structural data acquired with MRI and is employed to measure axonal integrity. It analyses the direction of water protons travelling along nerve fibers, thereby visualizing white matter nerve tract structure and orientation. In a diffusion tensor imaging study by Fernández-Espejo et al. [58], VS/UWS and MCS patients could be differentiated with 95% accuracy, based on structural brain damage in thalamus and subcortical white matter. Finally, magnetic resonance spectroscopy may be used for the detection of creatine, choline, N-acetylaspartate, and lactate in predefined regions of interest. These substances are considered to be biomarkers of aerobic energy metabolism, cell membrane synthesis/catabolism, cell viability/density, and anaerobic glycolysis, respectively. The ratios N-acetylaspartate/creatine and N-acetylaspartate/choline are currently being validated as prognostic markers in DOC patients [59, 60]. 

One of the main shortcomings of PET and fMRI is the relatively bad temporal resolution, which is generally in excess of 1.5 seconds. Cognitive processes usually take place on a much shorter timescale, in the order of milliseconds. An evaluation of remaining consciousness based only on PET or fMRI therefore lacks vital information. Electroencephalography is the method of choice to fill this niche in DOC diagnosis. 

### 3.3. Electroencephalography

Using “resting state” electroencephalography (EEG) can improve diagnosis and prognosis of DOC. Babiloni and colleagues [61] found that increased alpha power correlated with recovery in a group of VS/UWS patients. Similarly, occurrence of sleep spindles during periods of assumed sleep in DOC patients has been associated with better outcome [62]. In a recent EEG sleep study, all tested MCS patients showed characteristic sleep patterns resembling those of healthy subjects [63]. These included alternating periods of rapid eye movement and non-rapid eye movement sleep, as well as the phenomenon of shorter periods of slow-wave sleep at the end of the night than at the beginning of the night (this is a normal sleep development also occurring in healthy controls and thought to be related to neural plasticity). Few of such sleep patterns could be observed in VS/UWS patients. Instead, the VS/UWS patients showed EEG patterns that did not change from night to day or between eyes-open and eyes-closed recordings. However, other studies did show significant differences in EEG patterns during sleep as compared to wakefulness (for a review, see [62]). Gosseries et al. [64] showed that analysis of the entropy of resting state EEG data in patients in the (sub)acute phase (less than one month post injury) of either coma, VS/UWS or MCS also has possible differential diagnostic value. However, patient entropy measurements at more than one month post injury did not have this diagnostic potential.

By measuring “passive” event-related potentials using EEG, insight can be gained on how the brain reacts to salient external stimuli. In DOC patients, the occurrence of event-related potentials that are thought to be a result of cognitive functioning (e.g., P300 and mismatch negativity) [59] has been linked to an increased chance of recovery [65]. However, similar event-related potentials have been shown to occur in some chronic VS/UWS patients who failed to recover [66].

Schnakers et al. [37] have shown the usefulness of “active” event-related potential paradigms in DOC. Patients were instructed to count the number of times a target name was presented in an auditory stimulus train containing different names including the patient's own name (Figure 3). Nine MCS patients and none of the VS/UWS patients showed an increase in command-related event-related potentials after presentation of target names. This technique was also used to detect consciousness in a case of *total* locked-in syndrome [37], whereby a patient is fully conscious but completely unable to communicate by bodily movements or even eye-blinking. Cruse et al. [67] recently asked VS/UWS patients to move their hand and used EEG to measure the event-related potentials. These potentials were similar to normal in three patients, indicating that they were misdiagnosed as a result of their behavioral unresponsiveness.

Lastly, transcranial magnetic stimulation (TMS), in combination with EEG recording, can also be used for the assessment of residual brain function of DOC patients [68]. TMS is a method used to stimulate a region of the cortex, while EEG recordings make it possible to visualize changes in effective connectivity in response to this stimulation. Therefore, TMS-EEG can be used to analyze the intactness of neural circuits and can offer important clues about the state of consciousness a patient is in. TMS-EEG has been tested in healthy controls during midazolam-induced unconsciousness [69] and non-rapid eye movement sleep [70]. In these conditions, the cortical response following TMS remained more local and lasted for a shorter period of time than during wakefulness. Rosanova et al. [68] examined EEG responses to TMS stimulation in VS/UWS patients and detected short, localized responses similar to those seen in healthy controls during sleep and anesthesia. In contrast, the EEG response in MCS patients was more complex, travelled farther through the brain and lasted longer than in VS/UWS patients, more similar to the healthy awake state. 

### 3.4. Other Assessments

A variety of other diagnostic tools is currently in development and is being tested on DOC patients. Amongst the most promising are electromyography, “sniffing”-tests, and functional near-infrared spectroscopy (fNIRS). Electromyography has been used to study the occurrence of subthreshold muscle activity in response to verbal command. In a study by Bekinschtein and colleagues [71], one VS/UWS and two MCS patients showed increases in the electromyography signal related to the command “move your hand.” Willful modulation of nasal pressure (“sniffing”) can also be used for communication, writing texts and driving a wheelchair [72]. Sniffing can provide a control interface that is fast, accurate, robust and highly conserved following severe injury. It is therefore possible that this can be used as a diagnostic tool in DOC, although more research is needed. When studying the brain, fMRI has the advantage of showing with high precision the brain areas that are involved in cognition and consciousness. As mentioned before, this information can be used to communicate via brain modulation by the patient. However, although attempts for such fMRI-based communication have been successful in a number of cases [19], it has the disadvantage of being dependent on expensive and immobile fMRI scanning equipment. fNIRS might offer a solution to this problem in the near future [73], as it is a portable, silent, low-cost alternative to fMRI. The technique capitalizes on the changing optical characteristics of blood in the visible and near-infrared light range, when oxygenated hemoglobin in the blood becomes deoxygenated due to oxygen extraction by brain tissues. Although initial fNIRS studies have been performed in several neurological and psychiatric disorders [74], validation of the technique in DOC is still awaited. A limitation of fNIRS is the fact that it cannot measure activity in deep brain structures. However, the technique offers the possibility of continuous scanning for longer periods of time than would be possible with fMRI and can include patients that have physiological limitations that make fMRI scanning impossible.

## 4. Conclusion

After years of study, precise characterization of DOC remains elusive. In recent years, it has become ever clearer that the separate subconditions (coma, VS/UWS, MCS) fit into the percept of a gradually recuperating consciousness. With the help of standardized behavioral tests and PET scanning, it has become possible to subdivide the MCS into MCS− (i.e., nonreflex movements) and MCS+ (i.e., response to command). This raises questions regarding the phenomenon of “minimal” consciousness. When is minimal consciousness enough to call a patient conscious? What is the moment of “no consciousness” and how can we objectively measure this in another being? This problem is emphasized in the renaming of the vegetative state into unresponsive wakefulness syndrome, reminding physicians to remain careful when making inferences regarding conscious awareness based on behavioral assessment of motor responsiveness. 

Correct diagnosis of the level of remaining consciousness in a DOC patient is important for multiple reasons. First, it helps to ensure that appropriate caretaking can take place, tailored to the specific needs of each patient. These needs may include treatment for pain, and access to rehabilitation support and methods for motor-independent or motor signal-enhancing communication as mentioned in this review. Second, knowing the actual state of the patient can aid in prognosis. The experiments and their accompanying behavioral assessment, which was usually the CRS-r, mentioned in this review were performed from five days after brain injury to 23.7 years (Table 3). This shows that no general consensus exists on the time since injury at which to conduct differential diagnosis. However, the implications for palliative care and prognosis mean that behavioral assessment and neuroimaging should be administered as soon as the patient is stabilized and shows signs of brain arousal (i.e., recovery from the comatose state). As the condition of DOC patients is more prone to being transitional during the acute phase, caretakers should retest during the subacute phase. Third, the ongoing subcategorisation of DOC might have societal, ethical and legal consequences. Care of DOC patients is costly, and insurance companies might base payment of insurance money for this care on the presence of consciousness in the patient.

As for now, the CRS-r is still considered to be the best behavioral scale that exists for differential diagnosis of DOC. Its use in hospitals and nursing homes should be encouraged, as it has been shown to detect misdiagnosis at a rate of 30–40% [21], while at the same time being relatively easy to learn and apply by a member of the caretaking staff. A robust training in applying the scale and experience in conducting the scale is definitely recommended. Furthermore, it should be noted that the trust in the CRS-r to deliver good differential diagnosis is not based on validation with a gold standard, as no such standard exists. Rather, credibility for the differential capabilities of the scale comes from proven good interrater reliability, test-retest reliability, internal consistency, the fact that it includes all of the Aspen Workgroup criteria for good standardized administration and scoring, as well as its consistency with neuroimaging results. 

Brain imaging techniques based on passive paradigms, as well as other ancillary methods, are being validated for accuracy. The increasing number of reported success stories in recent literature illustrates the importance and improvements of these techniques. PET scanning is one of the most valuable additional tests at the moment, having shown to be able to make a probabilistic distinction between unconscious (VS/UWS) and conscious (locked-in syndrome and controls). Active paradigms, however, can have increased diagnostic power augmenting that of behavioral tests and passive paradigms. Mental imagery fMRI or EEG paradigms, such as those where a patient is asked to think of playing tennis or walking through the house to answer “yes” or “no,” can be validated by asking questions to which the answer is known. This technique, and variants of it, have already been successfully used in clinical practice. Only limited inferences can be made on the quality of the conscious experience in a patient in which consciousness has been detected according to neuroimaging-based examinations, but not according to behavioral tests. An intact DMN increases the chance of the patient having spontaneous, self-reflective thoughts (considered to be a key ingredient of human consciousness); brain responses to stimuli as seen by PET, fMRI or EEG that seem near-to-normal suggest that the patient can “feel” the stimulus. Our closest approximation to proof of cognitive functioning similar to conscious control subjects comes from those patients that manage to willfully modulate brain activity or even answer to “yes”/“no”-questions. However, it is questionable to what extend MCS patients can answer difficult questions regarding self-reflection correctly. Learning more about the subjective experience of minimal, fluctuating consciousness in MCS patients might therefore be extremely difficult using question-answer paradigms. Extrapolating insight into the self-reflection capability of MCS+ patients or those patients that have exited the MCS might be one strategy.

Contrary to standardized behavioral examinations, the high acquisition and maintenance costs of PET and fMRI machines, as well as the necessary availability of experienced staff to scan the patient and analyze and interpret the scanning data, place them out of reach for many centers. These costs will eventually go down, while up-to-date methods of analysis of brain scanning data, obtained in a nonspecialized medical center with enough expertise to conduct a specific ancillary test, might be performed by specialized coma centers. Furthermore, the development of cheaper, portable and easier to apply techniques, like those based on EEG and fNIRS, seems to offer promising alternatives to fMRI. With appropriate though relatively limited training, these techniques could even be used in nursing homes or at the patient's home (although application and data analysis of the method might still be done by specialized researchers), thereby reaching a bigger group of patients. Even though the use of ancillary diagnostic techniques in the acute phase is usually limited to acquisition of structural (MRI/computer tomography) brain images, stabilized subacute and chronic patients can benefit from the increasing scale of ancillary diagnostic methods described in this paper. 

## Figures and Tables

**Table 1:** Coma Recovery Scale revised.

	Description	Scored when
Auditory function		

(4) Consistent movement to command^#^	Object-related eye or limb movement or movement with nonobject-related commands.	On all 4 trials on 2 different commands.
(3) Reproducible movement to command^#^	Object-related eye or limbs movement or movement with nonobject related commands.	3/4 trials on any one of the object or nonobject related commands.
(2) Localization to sound	Auditory stimulation (e.g., voice or noise) from the right and the left side for 5 s. Repeat the procedure 2 times on each side.	3/4 trials on any one of the object or nonobject related commands.
(1) Auditory startle	Auditory stimulation directly above the patient's head and out of view (4 trials).	Eyelid flutter or blink following the stimulus on at least 2 trials.
(0) None	Observed response to above method.	No response to any of the above.

Visual function scale		

(5) Object recognition*	Object-related eye or limb movement commands.	3/4 clearly discernible responses.
(4) Object localization: reaching*	The patient is asked to touch an object with his/her arm or leg, 4 trials (2 left, 2 right presentations).	Movement must occur in the correct direction on 3/4 trials.
(3) Pursuit eye movements*	Move mirror to the right, left, upper, and lower directions. 2 trials in every direction (manually open eyes if necessary).	Eyes must follow the mirror for 45 degrees without loss of fixation on 2 occasions in any direction.
(2) fixation^∧^	Present a brightly colored object in front of the patient's face and then rapidly move to upper, lower, right, and left visual fields for a total of 4 trials (manually open eyes if necessary).	Eyes change from initial fixation point and then fixate on the new target location for more than 2 s. At least 2 fixations.
(1) Visual startle	Quickly move a finger to 1 inch in front of the patient's eye, while avoiding contact with the eyelashes or inadvertent production of a breeze (manually open eyes if necessary). 4 trials per eye.	Blink promptly following presentation of visual threat on at least 2 trials with either eye.
(0) None	Observe response to above method.	No response to any of the above.

Motor function scale		

(6) Functional object use^+^	Place one object (comb) in the patient's hand and instruct the patient to ‘‘show me how to use it.” Repeat the same instruction with a second object. 2 trials for each object.	Movements executed are compatible with specific function for both objects on all 4 trials.
	Observe for spontaneous automatic motor behaviors (nose scratching, grasping bedrail) during the examination.	At least 2 episodes of automatic motor behaviour are observed within the examination.
(5) Automatic motor response*	or present a familiar gesture (e.g., wave) and ask the patient to ‘‘show me how to wave” 2 times and ‘‘I'm going to wave again. Do not move at all. Just hold still” 2 times (alternate each command)	Patient performs the gesture on trials ‘‘just hold still.”
	or place a spoon in front of the patient's mouth without making contact and ask the patient to ‘‘show me how to use the spoon” 2 times and ‘‘I'm going to show you a spoon. Do not move at all. Just hold still” 2 times.	Patient performs the gesture on trials ‘‘just hold still.”
(4) Object manipulation*	Place a ball on the dorsal surface of the patient's hands and roll the ball across the index finger and thumb without touching the surface of the hand or fingers. Instruct the patient to ‘‘Take the ball.” 4 trials.	3/4 trials, the wrist must rotate and the fingers should extend as the object is moved along the dorsal surface of the hand; the object must be grasped and held for a minimum of 5 s.
(3) Localization to noxious stimulation*	Apply deep pressure to nail beds of extremities for a minimum of 5 s. 2 trials on each side for a total of 4 trials.	The nonstimulated limb must locate and make contact with the stimulated body part at the point of stimulation on at least 2/4 trials.
(2) Flexion withdrawal	Apply deep pressure to nail beds of each extremity. 1 trial per extremity.	Isolated flexion withdrawal of at least 1 limb.
(1) Abnormal posturing	Observe response to above method.	Slow, stereotyped flexion or extension of the extremities immediately after the stimulation.
(0) None/flaccid	Observe response to above method.	No response to any of the above.

Oromotor/verbal function scale		

(3) Intelligible verbalization^#^	Ask the patient to answer autobiographical or object naming questions.	Each verbalization must consist of at least 1 consonant-vowel-consonant triad, and 2 different words must be documented, and words produced by writing or alphabet board are acceptable.
(2) Vocalization/oral movement	Nonreflexive oral movements, spontaneous vocalizations or vocalizations that occur during administration of vocalization commands.	At least 1 episode of spontaneous nonreflexive oral movement and/or vocalization (yawning is scored as reflexive oral movement).
(1) Oral reflexive movement	Present tongue blade between patient's lips and/or teeth.	Clamping of jaws, tongue pumping, or chewing movement.
(0) None	Observe response to above method.	No response to any of the above.

Communication scale		

(2) Functional: accurate^+^	Ask 6 visual or auditory related questions (‘‘Am I touching my ear?”, ‘‘Am I clapping my hand?”).	Clearly discernible and accurate yes/no responses on all 6 of the visual or auditory related questions.
(1) Nonfunctional: intentional^#^	Observe response to above method	Clearly discernible and accurate yes/no responses on at least 2/6 of the visual or auditory related questions.
(0) None	Observe response to above method	No discernible verbal or nonverbal communication.

Arousal scale		

(3) Attention	Consistency of behavioral responses following verbal or gestural prompts.	No more than 3 occasions across the length of the evaluation in which the patient fails to respond to a verbal prompt.
(2) Eye-opening w/o stimulation	Observe status of the eyelids across length of assessment.	Eyes remain open across the length of the examination without the need for any stimulation.
(1) Eye-opening with stimulation	See above.	Tactile, pressure, or noxious stimulation must be applied at least once during the examination in order for the patient to sustain eye opening.
(0) Unarousable	See above.	No eye opening.

^∗^denotes MCS−.

^#^denotes MCS+.

^+^denotes emergence from MCS.

^*∧*^denotes an MCS except for anoxic etiology.

**Table 2:** The Nociception Coma Scale revised.

	Description
Motor response	

(3) Localization to noxious stimulation	The nonstimulated limb must locate and make contact with the stimulated body part at the point of stimulation.
(2) Flexion withdrawal	There is isolated flexion withdrawal of at least one limb. The limb must move away from the point of stimulation.
(1) Abnormal posturing	Slow, stereotyped flexion, or extension of the upper and/or lower extremities occurs immediately after the stimulus is applied.
(0) None/flaccid	There is no discernible movement following application of noxious stimulation, secondary to hypertonic or flaccid muscle tone.

Verbal response	

(3) Verbalization (intelligible)	Production of words in response to nociceptive stimulation. Each verbalization must consist of at least 1 consonant-vowel-consonant (C-V-C) triad. For example, “aie” would not be acceptable, but “stop” or “that hurts” would.
(2) Vocalization	At least one episode of nonreflexive oral movement and/or vocalization in response to stimulation (such as “ah” or “aie”).
(1) Groaning	Groans are observed not spontaneously but in response to nociceptive stimulation.
(0) None	No response to any of the above.

Facial expression	

(3) Cry	Cries are observed not spontaneously but in response to nociceptive stimulation.
(2) Grimace	Grimaces are observed not spontaneously but in response to nociceptive stimulation.
(1) Oral reflexive movement/startle response	Clamping of jaws, tongue pumping, yawning, chewing movement.
(0) None	There is no discernible facial expression following application of noxious stimulation.

**Table 3:** Overview of ancillary assessments.

Study	Type	Behavioral scale	Time between brain injury and assessment
Thibaut et al. [36]	PET	CRS-r	17 days–270 months
Phillips et al. [38]	PET	CRS-r	1–285 months
Bruno et al. [7]	PET	CRS-r	1.2–82 months
Boly et al. [39]	PET	GCS	20–124 days
Boly et al. [12]	PET	GCS	37–116 days
Laureys et al. [42]	PET	GCS	36 ± 9 days
Vanhaudenhuyse et al. [45]	fMRI	CRS-r	5 days–5 years
Qin et al. [52]	fMRI	CRS-r	2–18 months
Rodriguez Moreno et al. [54]	fMRI	CRS-r	2 months–2 years
Bekinschtein et al. [55]	fMRI	CRS-r	5 and 16 months
Monti et al. [19]	fMRI	CRS-r	1.3–60.8 months
Bardin et al. [57]	fMRI	CRS-r	—
Fernández-Espejo et al. [58]	fMRI	CRS-r	1–19 months
Babiloni et al. [61]	EEG	—	32–98 days. Follow-up was done after 3 months
Landsness et al. [63]	EEG	CRS-r	25 days–25 years
Gosseries et al. [64]	EEG	CRS-r	<1 month
Cavinato et al. [65]	EEG	—	2-3 months
Fischer et al. [75]	EEG	—	4–261 months. Follow-up was done for up to 1 year
Perrin et al. [66]	EEG	CRS-r	13 days–10 months
Schnakers et al. [37]	EEG	CRS-r	12 days–23.7 years
Rosanova et al. [68]	TMS-EEG	CRS-r	12–1399 days
Bekinschtein et al. [71]	EMG	CRS	3 or more months

PET: positron emission tomography; fMRI: functional magnetic resonance imaging; CRS-r: Coma Recovery Scale revised; GCS: Glasgow Coma Scale [76]; CRS: Coma Recovery Scale.
